# Patterns in Marine Fungal Diversity and Community Structure on Native Versus Invasive Macroalgae at a Local Geographic Scale

**DOI:** 10.1007/s00248-026-02781-8

**Published:** 2026-04-23

**Authors:** Elizabeth A.M. Owen, Robert I. Griffiths, Peter N. Golyshin, Tatyana N. Chernikova, Martyn Kurr

**Affiliations:** 1 Marine Centre Wales, School of Ocean Sciences, Isle of Anglesey, Bangor University, Menai Bridge, LL59 5AB UK; 2 Environment Centre Wales, School of Environmental and Natural Sciences, Bangor University, Bangor, LL57 2UW UK; 3 Centre for Environmental Biotechnology, School of Environmental and Natural Sciences, Bangor University, Bangor, LL57 2UW UK

**Keywords:** Marine Fungi, Macroalgae, Microbiome, Invasive, *Sargassum muticum*, *Fucus serratus*

## Abstract

Nonindigenous marine species are a growing threat to coastal ecosystems, but invasive dynamics are understudied in marine environments. The microbiome of invasive macroalgal species has been recognised as contributing to their invasive success, but research to date has largely focussed on the prokaryotic fraction. This research compared the epiphytic fungal communities between a native (*Fucus serratus*) and an invasive (*Sargassum muticum*) macroalga, across a relatively small spatial scale (< 800 m), in North Wales. DNA extracts from surfaces of macroalga were PCR-amplified with primers ITS1F and ITS2 and sequenced using Illumina MiSeq. Difference in fungal community composition was observed between all sample groups with the exception of *S. muticum* and *F. serratus* samples taken from within a few meters of each other, suggesting environmental drivers of the macroalgal fungal community are more important than host specificity. It also raises the question of whether *S. muticum* exerts a direct influence on the microbiome of neighbouring algal species, although further research is required to explore this potential interaction. No differences were detected in fungal biodiversity across any of the samples, supporting prior findings of a natural peak in fungal diversity at local scales.

**Supplementary Information:**

The online version contains supplementary material available at 10.1007/s00248-026-02781-8.

## Introduction

Macroalgae are key species in coastal ecosystems, being photosynthetic, primary producers, and a food source, habitat, nursery ground, and refuge for coastal organisms [1]. Canopy forming macroalgae moderate the risk of natural disasters through the reduction of coastal wave force, and erosion [2]. With a global annual net primary production of 0.13–2.9 Pg of carbon, macroalgal stands are amongst the most productive habitats [3] and contribute to the sequestration of 61–268 Tg of carbon per year [4]. Economically, they are important in bioremediation, nutraceuticals, pharmaceuticals, and are an important and growing global food source [5].

Coastal ecosystems and their associated macroalgal assemblages are increasingly threatened by the impacts of climate change and anthropogenic-related stressors [6], including invasive species [7]. Invasive marine algae, estimated to compromise up to 40% of global non-indigenous species [8], are spreading rapidly via aquaculture and shipping, floating plastic debris, and migratory marine fauna [7]. Invasive success is likely the result of a complex set of biotic and abiotic traits and interactions that support invasive macroalgal defences when in a new environment and under high biological pressure [9]. The flexibility of non-native algae to rapidly shift their chemical profile in response to new environments may enhance their invasive success, and macroalgal self-regulation of secondary metabolites is associated with control over its microbiome [9].

Microbes are essential for the growth, development, and ultimate decay of macroalgae [10–12], with the health and overall performance of an alga being strongly linked to active mutualistic interactions with its microbiome [13]. Seaweed associated epiphytic microbial communities are involved in host nutrient provisioning, anti-microbial production, and biogeochemical cycling, contributing to their successful adaption and resilience to environmental stressors and gradients [13].

Previous research has concentrated on the bacterial fraction of the microbiome, with historical exploration of the eukaryotic fungal contribution being reliant on culture-based approaches. 16 S rRNA bacterial communities of *Sargassum* spp. have been examined across multiple locations, revealing variability in microbial composition [14, 15]. Significant differences in prokaryotic community structure and bacterial selectivity of *Sargassum* compared with surrounding seawater, have also been reported [15].

There is no single unifying mechanism underlying invasion success; rather, it likely results from a complex interplay of biotic and abiotic factors. Beyond classical invasion theories such as the Novel Weapon and Shifting Defence Hypotheses, which emphasise biochemical defence strategies, increasing evidence suggests that microbiome flexibility may also contribute to invasive performance [16]. Macroalgae regulate surface-associated microbial communities through metabolite-mediated selection, influencing epibiont composition, host health and resistance to fouling or pathogens [9, 17]. In *Agarophyton vermiculophyllum*, invasive populations were shown to exhibit greater tolerance to microbial disturbance and increased microbiome convergence when exposed to novel microbial environments, over native populations [16]. This suggests reduced dependence on specific symbionts and an enhanced capacity to restructure host-associated communities, mechanisms likely shared by other invasive algae, including *Sargassum muticum* [18]. Such “host promiscuity” may facilitate rapid acclimatisation to novel microbial landscapes, thereby promoting invasion success under shifting environmental and biological conditions.

Variations observed in the epiphytic microbial community of invasive *A. vermiculophyllum*, across geographical space, suggest that community composition is impacted by local, and potentially wider, abiotic factors [9]. Conversely, invasive algal species have shown dominant fungal variants not associated with sympatric native algal species or surrounding sea water, suggesting host taxonomy or phylogeny as a driver of fungal diversity [10]. Fungal community composition has also been shown to differ across tissue type (leaves, holdfasts, and vesicles) in *Sargassum ilicifolium* [19], as well as between apices, mid-thallus, and stipe, in the closely-related fucoid algal species *Sargassum muticum*, *Pelvetia canaliculata*, and *Himanthalia elongata* [10]. Species richness was shown as being greater on older tissues [10].

2,310 marine fungal species have been recorded, but only 50% have associated molecular data and only 64 have been sequenced [20]. Isolates have been obtained from diverse substrates and habitats, including mangroves, driftwood, coral, sediments, and seawater [20]. Macroalgae in particular are well known to host symbionts, saprotrophs, and parasites from the Ascomycota, Basidiomycota, Chytridiomycota, and Labyrinthulomycota, although many taxa are identifiable only to genus level [20, 21], and communities are dominated by a small number of cosmopolitan genera (Aspergillus, Penicillium, Fusarium, Chaetomium, Alternaria, and Cladosporium) [22]. Research has largely focused on endophytic fungi within algal cells, whereas biofilm-associated epiphytic fungi remain poorly studied. Many marine fungal species are yet to be classified [23], and their host specificity, environmental drivers, and ecological functions remain poorly understood [24].

This research aimed to compare epibiotic fungal community composition between an invasive and a native macroalga, from the same locality. Samples of the non-native *Sargassum muticum* (Yendo) Fensholt 1955 and the native *Fucus serratus*, Linnaeus, 1753, were collected from a site in North Wales, known to have been invaded by *S. muticum* at least 25 years prior [17]. Differences in fungal community composition, patterns of diversity and relative abundance were determined using Illumina next-generation sequencing, and compared to those in the surrounding seawater and sediment. *Sargassum muticum*, native to Japan and first recorded in the UK in 1973 [25], is a particularly impactful invasive brown alga, outcompeting native, slower growing, canopy forming fucoids [21], and acting as an ecosystem engineer [26, 27]. *Fucus serratus* is typically sympatric with *S. muticum* in the UK and is a useful native comparison in ecological studies [28]. Seawater and sediment samples were collected to serve as background and environmental comparators for microbial composition.

It was hypothesised that: (1) fungal diversity and community composition would differ between macroalgal species, regardless of macroalgal spatial proximity, and despite expected consistency in environmental variables across sites; (2) fungal diversity and community composition would differ between algae and seawater; and (3) fungal diversity and community composition between sediment samples would not differ, due to assumed similarities in environment between sites.

## Methods

### Fieldwork Methods and Sampling

Two sites (5 m^2^ each, and approximately 800 m apart) in the lower eulittoral zone on the southern shore of the Menai Strait, Wales, UK, were selected. Site A (53.132444, -4.306361) contained both *Sargassum muticum* (invasive) and *Fucus serratus* (native), whereas Site B (53.126861, -4.314000) contained only *F. serratus*. Site B, with no known history of *S. muticum* invasion, was chosen to evaluate the effects of this invasive species on the fungal community composition of the native macroalga *F. serratus*. The substrate at Site A was muddy silt with an abundance of cobbles around 3–5 cm in diameter; Site B was somewhat sandier, and had notably fewer cobbles. Site B was also around 10 m further up the shore, making it approximately 50 cm higher above chart datum. Aside from these relatively small differences, the two sites were broadly similar. Ideally, the two sites would have been identical in substrate composition and tidal height, but *S. muticum* was present at all such sites within 1 km from Site A and Site B was chosen as the best compromise. The tidal range in the Menai Strait is around 6 m, making a 50 cm difference in vertical height relatively small. Typical intertidal macroalgae were present at both sites, including *Palmaria palmata*, *Ulva lactuca*, *Himanthalia elongata*, *Fucus spiralis*, *Chondrus crispus*, *Halurus flosculosus*, and *Laminaria saccharina*; although relative abundances of each were different between the sites and Site A was clearly the more biodiverse. Samples were collected on the same day, at low tide in the month of May, 2023, during *S. muticum*’s active growth period.

Ten samples of each target species were collected from Site A, and ten *F. serratus* from Site B. Holdfasts were chosen for sampling, as a stable, persistent macroalgal structure. In *Sargassum muticum*, holdfasts are perennial whilst fronds are seasonally regenerated; similarly, in *Fucus serratus*, the holdfast and basal thallus persist between years. Holdfasts were removed using a shucking knife and placed in zip lock bags. Samples ranged from 2 to 5 cm in length. Five sediment samples per site were collected randomly, and larger cobbles (> 1 cm) were excluded from collection. Nitrile gloves were worn throughout to limit contamination of the samples. Seawater samples (3 × 1 L) were collected in autoclaved carboy bottles from the Menai Strait adjacent to the sampling locations. It was not deemed necessary to collect water from Sites A and B separately because the tidal flow in this part of the Strait is typically around 1.5–2.5 ms^− 1^ [29, 30], and so the ambient seawater is essentially the same between them. Water passing over Site A then passes over Site B within a few minutes at ebb tide and vice versa during the flood, twice per day.

### Sample Preparation

Seawater and algal samples were stored at 4 °C and processed within 72 h. From each seawater sample, 750 mL was filtered (0.2 μm cellulose acetate filters) and frozen at − 20 °C. Algal holdfasts (1–5 cm, 0.3–6.8 g) were excised, washed (vortexed for 30 s) in 10 mL ONR7a medium, and centrifuged 2 ml at a time (5× at 18,000 g, 10 min, 10 °C) to pellet microbial biomass. On the fifth and final step, 1800 µL of the supernatant was removed and the aggregated pellet was resuspended in the 200 µL of supernatant, and stored at − 20 °C. Sediment samples were stored at 4 °C for < 7 days prior to DNA extraction, without the requirement of advance preparation.

### DNA Extraction

DNA extraction was undertaken using the Qiagen DNeasy^R^ PowerLyzer^R^ PowerSoil^R^ Kit following the standard protocol provided (Qiagen, 2020). The Fisherbrand Bead Mill 24 was used for homogenisation, with modifications: (2 cycles of 2 min, at 2.4 m/s, 10 s dwell between cycles) and 80 µL of elution buffer solution C6 (vs. 100 µL standard).

DNA of the yeast *Saccharomyces cerevisiae* was extracted for use as a positive control.

DNA concentrations were measured with a Fluorometer (Qubit 4, Invitrogen), using the Qubit 1X dsDNA High Sensitivity Assay kit (Invitrogen, UK). Final Qubit volume equalled 200 µL, with 5 µL of extracted DNA added to 195 µL of Working Solution. Remaining extracted DNA was stored at -20 °C. Concentrations of DNA extracted from water samples ranged from 0.84 to 1.59 ng/µL, and from sediment samples from sites A and B within 6.20–11.20 ng/µL and 4.00-13.40 ng/µL, respectively. *S. muticum* and *F. serratus* DNA concentrations across samples from site A varied from 0.04 to 6.16 ng/µL. and 0.40–17.10 ng/µL, respectively. DNA concentrations extracted from *F. serratus* at site B were within the range of 0.04–12.70 ng/µL, with a positive control (*S. cerevisiae*) of 4.56 ng/µL.

### PCR Amplification and Purification

The Internal Transcribed Spacer (ITS) 1 region was targeted and amplified using an ITS1F (5’-CTTGGTCATTTAGAGGAAGTAA-3’) [31] and ITS2 (5’-GCTGCGTTCTTCATCGATGC-3’) [32] primer pair. The PCR mix was prepared in a total volume of 25 µL, containing 7 µL molecular-grade water, 10 µL 2× Brilliant III SYBR Green qPCR Master Mix (Agilent, USA), 0.5 µL each of forward and reverse primers (10 µM; Eurofins, UK), and 2–10 ng extracted DNA. Negative control contained molecular-grade water in place of template DNA, and positive control contained 4.56 ng extracted DNA extracted from *Saccharomyces cerevisiae.*

PCR was completed using the AriaMx RT-PCR System (Agilent, USA) and the following conditions applied: 95 °C for 3 min, followed by 35 cycles of 95 °C for 10 s, 55 °C for 30 s, and 72 °C for 1 min, concluding with a final elongation step of 72 °C for 5 min.

After amplification of ITS 1 region, each sample was barcoded with unique index primers. PCR mix was prepared in a volume of 30 µl and contained 8 µL molecular grade water, 15 µL of 2 x concentrated Brilliant III SYBR-Green qPCR Master Mix (Agilent, USA), 1.5 µL each of a forward and reverse uniquely barcoded primer (10 µM; Eurofins, UK), and 4 µL of PCR product from the previous PCR. Thermal cycling programme for this step consisted of 3 min at 95 °C, followed by 15 cycles of 95 °C for 25 s, 56 °C for 20 s, and 72 °C for 30 s, with final elongation of 5 min at 72 °C.

Amplicons were visualised on 1.2% agarose gel stained with SafeView Nucleic Acid Stain (NBS Biologicals Ltd, UK). Gels were run at 90 volts for 30 min and visualised on a transilluminator (Invitrogen). PCR products (~ 400–600 bp) were excised and purified using the NucleoSpin Gel and PCR Clean-up Kit (Macherey-Nagel, Germany) following the standard protocol. Purified barcoded amplicons were quantified using a Qubit 4 (Invitrogen), and stored at − 20 °C. After the final library preparation and pooling, the final concentration of the pooled library, prior to sequencing was 4–16 pM for final loading into the MiSeq system.

Sequencing of barcoded amplicons was completed using the Illumina MiSeq platform (Illumina Inc., San Diego, CA) using 500-cycle v2 chemistry (2 × 250 bp paired-end reads) at the Centre for Environmental Biotechnology, Bangor, UK. Final analysis included the following samples: 9 x *F. serratus* and 9 x *S. muticum* from Site A, 8 x *F. serratus* from Site B, 5 x sediment from each site, 3 x seawater.

### Bioinformatics and Data Analysis

Raw ITS amplicon sequences were processed using the DADA2 pipeline in R. Reads were quality filtered, trimmed, denoised, merged, and chimeras removed to generate amplicon sequence variants (ASVs). Taxonomy was assigned using the UNITE fungal reference database (version 10.0 2024-04-04) via the assignTaxonomy function, using a confidence threshold of 70%. Raw data (number of reads per ASV across samples) was transformed into relative abundance to account for variance in sample size. Univariate and multivariate statistical analyses were performed on RStudio 4.2 and PRIMER7, respectively. All plots were created using RStudio 4.2.

Univariate measures of community structure (Shannon-Wiener and Simpson indices) were calculated on relative abundance data, and assessed for normality (Shapiro-Wilk), and homogeneity of variance (Bartlett or Levene). One-way analysis of variance (ANOVA) was performed on Shannon-Wiener (H’), and non-parametric Kruskal-Wallis on Simpson (1-λ) diversity indices.

**Fig. 1:**
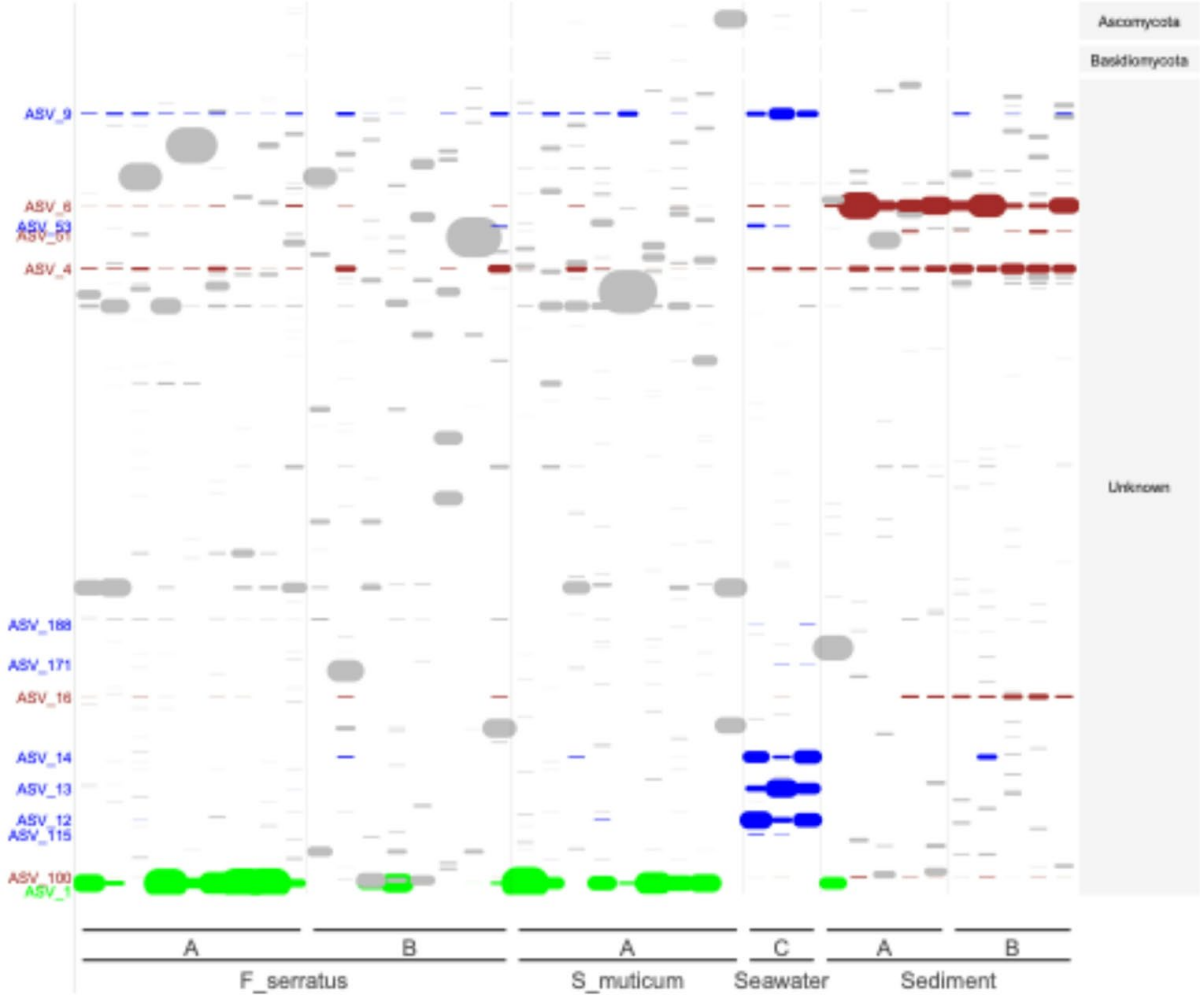
Gel plot highlighting indicator species (ASVs) and relative size of effect of individual ASVs across algae, seawater and sediment. ASV ID is defined on the left panel and associated phyla on the right. Along the bottom is associated host (*Fucus serratus*, *Sargassum muticum*, Seawater and Sediment) and the site from which samples were taken (Site A or Site B). Site C refers to Seawater only

Species richness was plotted against Shannon and Simpson diversity with Pearsons and Spearman’s rank correlation tests performed, respectively, depending on whether parametric assumptions were met. Indicator species responding significantly to treatments were identified using the indval function from the labdsv R package [33]. PRIMER7 was used to produce a Bray-Curtis dissimilarity matrix from which a non-metric multidimensional scaling (nMDS) plot was created. Multivariate Analysis of Similarity (ANOSIM) was performed to compare fungal community composition between algal, substrate, and seawater types, and Similarity Percentage (SIMPER) analyses showed which ASVs contributed most to within group similarity and between group differences. The top 15 ASVs contributing to between algal group difference (*F. serratus* A and *S. muticum* A; *F. serratus* A and *F. serratus* B; *S. muticum* A and *F. serratus* B) were evaluated for both normality and homogeneity of variance, and univariate statistical analysis performed via ANOVA or the Kruskal-Wallis test, followed by Tukey’s HSD or Dunn’s post hoc tests, respectively. Code used to generate Fig. 1 is available in a public GitHub Gist: https://gist.github.com/robiwangriff/234074587a5778e67862fd98989146d1.

## Results

A total number of 244,624 raw reads were generated from the sequencer. Following quality control and denoising through the dada 2 pipeline a total of 81,089 reads remained across 301 dereplicated ASVs, including the positive control. Of the sequences extracted, eight were assigned to the phyla *Ascomycota* (2.7%) and four to *Basidiomycota* (1.3%). All other ASVs were unassignable (95.7%), with the exception of the positive control, *Saccharomyces cerevisiae.* Full ASV counts and taxonomic assignments are reported in Table S1. Rarefaction analysis indicated that sequencing depth was sufficient to capture the majority of the observed diversity, as curves approached an asymptote across samples (Figure S1). Mean ASV richness across samples and total ASV counts per group are summarised in Table 1. Three ASVs occurred in all hosts, and 17 ASVs were detected in all macroalgal host groups (Table S1).

**Table 1:** Mean ASV richness per host group and total ASV counts

Host Group (Site)	Mean ASV across samples (SD)	Total ASVs within each host group
*Fucus serratus* (A)	19.11 (8.75)	89
*Sargassum muticum* (A)	18.0 (8.93)	104
*Fucus serratus* (B)	14.13 (4.94)	78
Sediment (A)	12.4 (1.14)	41
Sediment (B)	19.8 (3.83)	60
Seawater	18.0 (9.0)	35

**Fig. 2:**
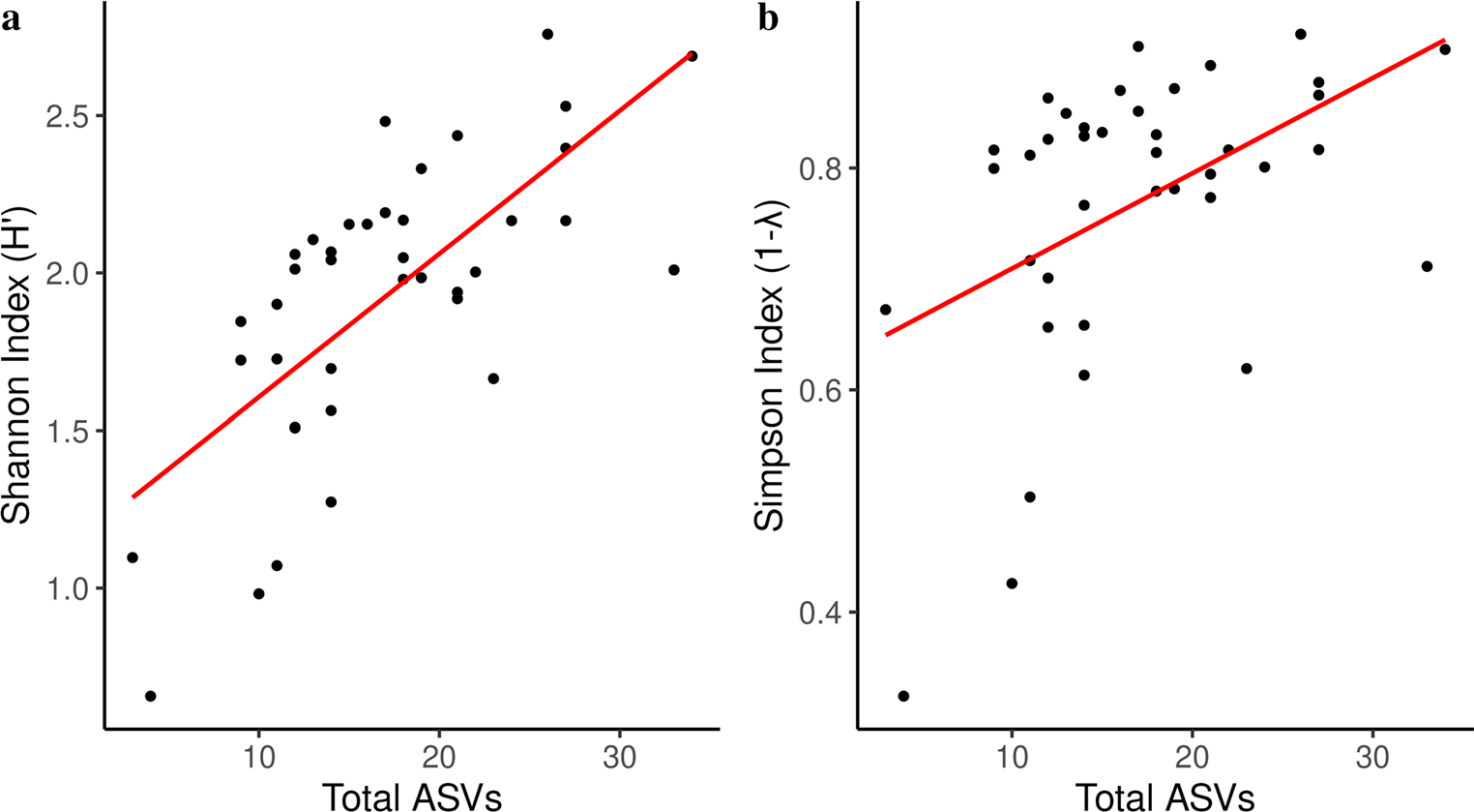
Increase in diversity with an increase in ASV richness. Significant positive correlation found between (**a**) Total ASV against Shannon-Wiener indices (Pearson’s correlation; t = 5.939, df = 37, *n* = 39, *r* = 0.699 (95% CI: 0.49, 0.83), *p* < 0.001) and plot (**b**) Total ASV against Simpson (Spearman’s Rank; *n* = 39, ρ = 0.446, *p* < 0.001)

Indicator ‘species’ (ASVs) across algae, seawater and sediment were highlighted (Fig. 2) with ASV1 being the strongest reflector of algal species. ASVs highlighted as being most strongly associated with seawater include ASVs 9, 12, 13,14, 53, 115, 171 and 188. Sediment is indicated by ASVs 4, 6, 16, 51, 71 and 100. A search through the NCBI (National Centre for Biotechnology Information, 1988) core nucleotide BLAST [34] database of key ASVs showed the majority of nucleotide matches were too short to be reliable, and where matches were available, they were largely uncultured fungal or eukaryote clones. ASV1 (KX113748.1) matched an uncultured planktonic eukaryote clone taken from marine surface water. ASV13 (MT000104.1) was matched as an uncultured fungus, isolated from filtered seawater in Swansea using ITSF and ITS2 primers, the same primers used in this research. A115 (KX115346.1), also matched an uncultured eukaryote clone isolated from marine surface water, and a match was found with ASV188 (JX974800.1) as an uncultured fungus clone from polluted estuarine sediment samples of Laizhon Bay, China.

## Biodiversity (Simpson and Shannon-Weiner)

**Fig. 3:**
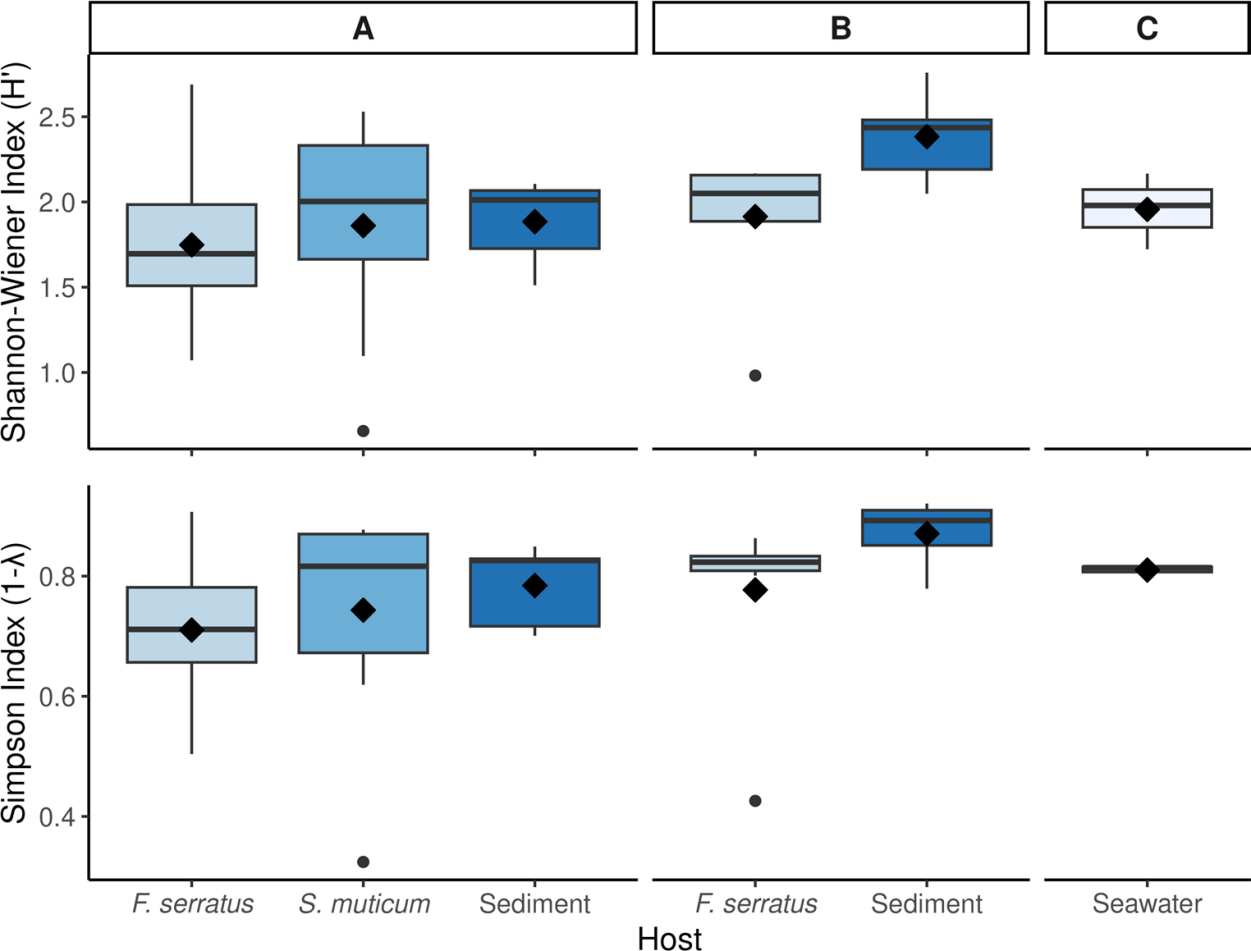
Biodiversity indices, (**A**) Shannon-Wiener rarified ASV richness, **B**) Simpson dominant ASV richness) of fungal community between groups separated by site. Site A (*Fucus serratus*, *Sargassum muticum* and sediment), invaded by *S. muticum*. Site B (*F. serratus* and sediment) free from *S. muticum*. Surrounding seawater also analysed (site C). No statistically significant differences present

There were significant positive correlations between species richness and the Shannon-Weiner (Pearson’s correlation; t = 5.939, df = 37, *n* = 39, *r* = 0.699 (95% CI: 0.49, 0.83), *p* < 0.001) and Simpson’s diversity indices (Spearman’s Rank; *n* = 39, ρ = 0.446, *p* < 0.001) indicating increasing diversity with increasing ASV richness (Fig. 2). Shannon-Wiener (ANOVA *n* = 39, F_5,33_=1.355, *p* = 0.266), and Simpson’s indices (Kruskal-Wallis, *n* = 39, *X*^2^ = 9.339, df = 5, *p* = 0.096), showed no difference in diversities between groups (Fig. 3).

### Community Composition

**Fig. 4:**
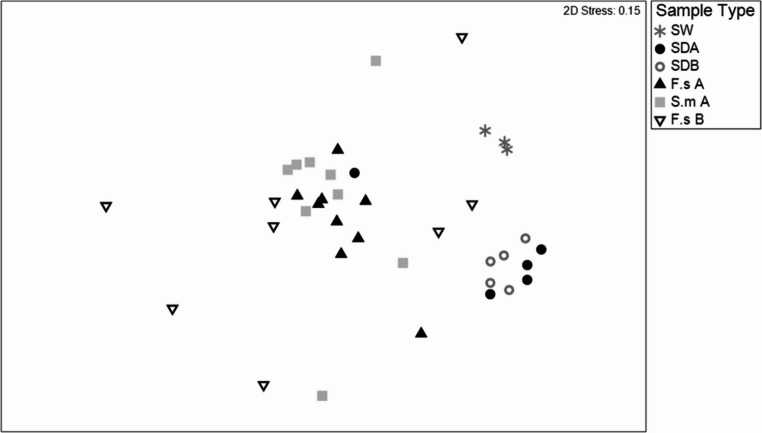
A non-metric Multi-Dimensional Scaling (nMDS) plot expressing the dissimilarity in fungal community structure between groups. (Sample Type=Group. SW=Seawater, SDA=sediment site A, SDB=sediment site B, F.s.A= *Fucus serratus* site A, S.m.A*= Sargassum muticum* site A, F.s.B = *F. serratus* site B)

A non-metric Multi-Dimensional Scaling (nMDS) plot (Fig. 4) indicates dissimilarity in fungal community structure between groups, with the exception of *S. muticum* and *F. serratus* at site A. *F. serratus* samples from site B show greater dispersion in community composition among within-group replicates. An Analysis of Similarity (ANOSIM) global test of difference (*R* = 0.415, *p* = 0.001), supported the observations of the nMDS, showing a high significance of difference in community composition between all groups except between *F. serratus* and *S. muticum* at site A (Table 2), suggesting that environmental variables drive fungal community composition. Significant differences in community composition were also seen between algae and sediment within each site, suggesting fungal composition in sediment is not the driver of macroalgal fungal community composition.

**Table 2:** Analysis of Similarities (ANOSIM) performed on relative abundance data indicating significant differences in community diversity between groups (SW=Seawater, SDA=sediment site A, SDB=sediment site B, *F.s.*A= *Fucus serratus* site A, *S.m*.A= *Sargassum muticum* site A, *F.s*.B = *F. serratus* site B)

Between group differences	*R* statistic	*p* value (* statistically significant)
SW, *F.s* A	0.858	0.005 *
SW, *S.m* A	0.500	0.009 *
SW, SDA	0.754	0.018 *
SW, *F.s* B	0.341	0.048 *
SW, SDB	1.000	0.018 *
*F.s* A, *S.m* A	-0.017	0.570
*F.s* A, SDA	0.758	0.002 *
*F.s* A, *F.s* B	0.307	0.001 *
*F.s* A, SDB	0.906	0.002 *
*S.m* A, SDA	0.597	0.002 *
*S.m* A, *F.s* B	0.237	0.007 *
*S.m* A, SDB	0.682	0.002 *
SDA, *F.s* B	0.370	0.026 *
SDA, SDB	0.276	0.032 *
*F.s* B, SDB	0.420	0.015 *

SIMPER analysis identified the percentage contribution of individual ASVs to the average within-group similarity. Analysis showed 26.44% within group similarity between individual samples of sediment at site A and sediment samples at site B had 42.46% within group similarity. Difference in fungal community composition between site sediment samples (ANOSIM, *R* = 0.276, *p* = 0.032) (Table 2) suggests potential environmental differences across site A and B, despite assumption of their broad similarity.

Investigation into algae only between sites A (*S. muticum* and *F. serratus* samples combined) and B (*F. serratus* only), continued to show significant differences in epiphytic fungal community composition (ANOSIM, *R* = 0.543, *p* = 0.001). SIMPER analysis showed an average similarity of 32.61% between algal samples at site A, and dominant ASVs (1, 3, and 2) remained consonant with analysis between sites when sediment samples were included. Average similarity between *F. serratus* samples at site B was 7.34% with dominant ASVs (percentage contribution to within-group similarity) being ASV10 (18.93%), ASV1(17.89%) and ASV11(12.68%). Between site dissimilarity (average: 89.54%) was mostly due to ASV1 (15.97%).

Proximity of seawater samples on an nMDS plot (Fig. 4) suggests a similarity in within group community structure. SIMPER analysis showed within group similarity of seawater samples to be 61.21% with ASVs 12, 13, 14, 9 and 4 attributed to 95% of within group similarity. Dominant ASVs in seawater are not the same as those found within other host groups.

**Fig. 5:**
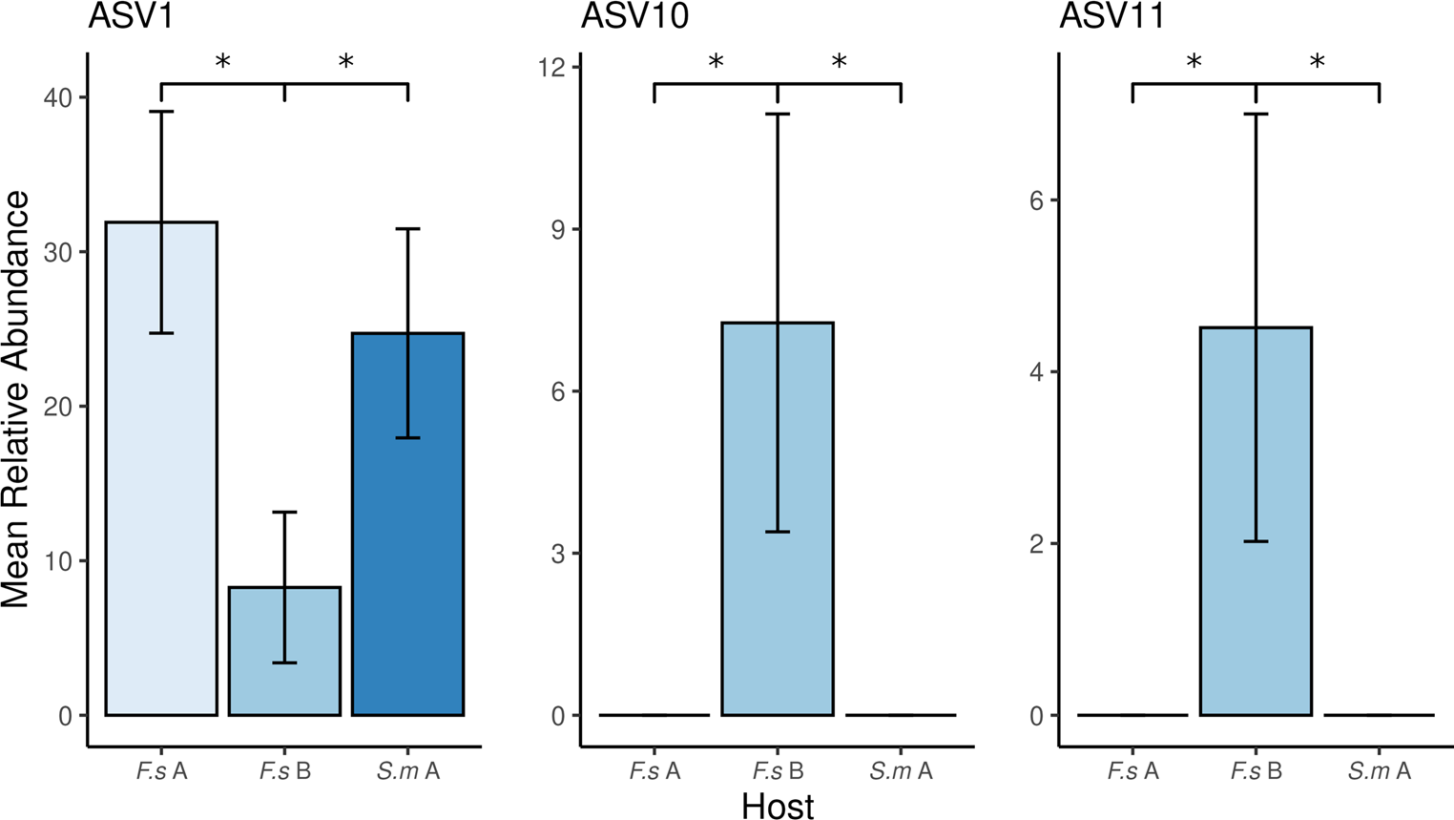
Significant difference in mean relative abundance found in three ASVs (ASV1, ASV10, and ASV11) of the top 15 individual ASVs contributing to difference between algal hosts (*F.s* A refers to *Fucus serratus* at site A; *F.s* B to *F. serratus* at site B; and *S.m* A to *Sargassum muticum* at site A). ASV 10 and ASV 11 were only present on *F. serratus* samples at site B

The top 15 ASVs contributing to algal group difference as determined by SIMPER output were compiled for statistical analysis (Tables S2a and S2b) in order to consider difference in presence across site and species. Three ASVs returned a significant result (Fig. 5).

## Discussion

This research suggests environmental factors are key contributors to the fungal community composition of *Sargassum muticum* and *Fucus serratus* within the same locality, in this case across sites separated by less than 1 km. Macroalgal fungal community composition differed between sites, including between the same species, but not across different species within the same site. This indicates a probable localised environmental influence on epiphytic fungal community composition. Observed inconsistency in sediment particle size, and difference in sediment fungal community composition between sites, suggests variation across local environmental factors. No difference in fungal diversity was seen across Shannon-Weiner and Simpson diversity indices between any groups. This suggests a potential consistency or peak in fungal diversity in samples within this environment.

The absence of significant differences in fungal diversity in this study aligns with other marine fungal research. No significant variation in Shannon-Weiner or Simpson indices was found across seven coastal sites along a 38 km Tunisian bay [35]. Similarly, no statistical difference was reported in fungal diversity across seven intertidal sites spanning 2,703 km in the U.S. Gulf of Mexico [36]. This pattern contrasts with diversity indices of marine macroscopic organisms, which typically show variation at small spatial scales due to complex ecological interactions such as resource availability, habitat changes, species dispersal, and recruitment [37, 38].

Comparative analysis of fungal diversity indices (Table S3) across this study, Nakbi et al. [35], and Walker and Robicheau [36] indicates consistent diversity despite varied sample substrates (macroalgae, sediment, seawater, seafoam, detritus, wood, and plastic debris) and geographies. Monastir Bay is a biodiversity hotspot classified as moderately to highly polluted [39], where the U.S. Gulf of Mexico is influenced by runoff from 33 major freshwater rivers and urban detritus from 31 cities. These considerations further support the suggestion of a threshold in marine fungal diversity, at least within a spatial scale < 3000 km, regardless of substrate or environmental variables.

However, it is important to interpret these similarities in light of methodological differences among studies. The present study employed a culture-independent, sequencing-based approach, which enables broader detection of fungal taxa but remains subject to sequencing biases. In contrast, Nakbi et al. [35] and Walker and Robicheau [36] relied on culture-based methodologies, which are inherently selective and tend to underestimate total fungal diversity by favouring fast-growing, readily culturable taxa. Despite these contrasting constraints, convergence of diversity indices across studies suggests that the observed patterns may reflect a genuine ecological signal; however, integrative approaches combining culture-dependent and culture-independent methods will be essential to fully resolve marine fungal diversity and validate the proposed diversity threshold.

A similar lack of difference in fungal diversity was reported across 11 macroalgal and 2 seagrass species in Northern China, using culture-independent methods, though this was not reflected in bacterial diversity [24]. Broader marine microbial ecology studies that have shown whole microbiome and prokaryotic communities vary significantly across environmental and geographic gradients [9, 40]. Previous investigation into the eukaryotic microbiome of *Agarophyton vermiculophyllum* found significant differences in both endo- and epiphytic communities across geographic scales, but did not specifically explore fungal communities [9]. Collectively, these results highlight the need to disaggregate microbial communities by phylogeny, distinguishing between bacterial, fungal, and other eukaryotic members, to better understand their diversity, recruitment mechanisms, and ecological roles in marine systems.

Differences in marine fungal community composition across sample sources, localities, and ecosystems are well documented [10, 19, 24, 36, 40], with major drivers of fungal biogeography including water temperature, salinity, oxygen availability, and substrate type [41]. Although abiotic conditions were assumed to be relatively consistent between sites owing to their close proximity (< 800 m) and similar morphology, observed differences in sediment fungal communities indicate the presence of local environmental heterogeneity even at this small spatial scale [42]. Broad-scale abiotic factors, including mean temperature, salinity, light exposure, and tidal flow, were likely comparable between sites, suggesting that observed variation is more plausibly driven by local-scale geomorphology and fine-scale tidal dynamics. Notably, Site B is situated closer to human development and the mouth of the Afon Seint (River Seint), which drains predominantly agricultural land and may represent a source of elevated nutrient inputs (e.g., nitrates and phosphates), potentially contributing to local variation in fungal community composition. Together with the significant differences in fungal community composition observed between all groups, except *S. muticum* and *F. serratus* at Site A, these findings suggest that small-scale environmental conditions possibly exert a stronger influence on fungal community structure than host identity alone.

Previous studies have observed an increased effect of environment on fungal community above that of the bacterial community [24], with fungal communities deemed to be less stable and less driven by host phylogeny. A study on the canopy forming macroalgae *Sargassum ilicifolium*, recorded significant differences in fungal community across 12 km [19], and macroalgal holobiont composition has been seen to vary both at local (population) and larger (hemisphere) geographical scales [9].

However, algal recruitment of microbes is not passive and has been shown to be a highly complex and dynamic process [24, 43, 44]. Indeed, it has been suggested that the ability of a host to recruit or selectively support specific microbes enhances its adaption to local environments [45], and even promotes invasive success [18].

Host identity has previously been proposed as the main driver of planktonic bacterial composition [46] and if specific host recruitment of microbes is a component of the macroalgal fungal community, the lack of difference in community composition between *F. serratus* and *S. muticum* at site A, combined with the significant difference in fungal community between *F. serratus* at site A and *F. serratus* at site B, could suggest the presence of *S. muticum* directly impacts the fungal community of other macroalgal hosts in its proximity, in this case *F. serratus.*

As a photosynthetic species in the epipelagic zone, *S. muticum* is vulnerable to fouling and predation. Defence mechanisms like epithallus sloughing; the shedding of its outer layer [47], branch fragility [28], senescence [48], and desiccation-induced tissue loss may facilitate microbiome dispersal to surrounding algae. Invasive *S. muticum* has been seen to alter the epifauna of native environments, through habitat and biodiversity modification [26, 27, 49]. The impact of invasive macroalgae microbiome on the microbiome of native macroalgae in its vicinity, however, is yet to be explored. The microbiome of invasive macroalga *Asparagopsis taxiformis* was found to alter the microbiome of the coral *Astroides calycularis* [50]. Changes in both microbial community and bioactive metabolites were observed both on non-contact proximity and on physical contact between hosts, with stronger host interactions leading to a reduction in inter-specific differences. Similarly, increased secondary metabolite production was reported in the native seagrass *Posidonia oceanica* in response to invasion by the macroalga *Caulerpa taxifolia*, indicating allopathic interactions [51].

Among the ASVs assigned to *Ascomycota*, two were classified to family level: *Cordycipitaceae*, a predominantly entomopathogenic but ecologically adaptable family (order *Hypocreales*) [52] with marine-associated strains reported from Antarctic macroalgae [53] and as endophytes of the brown alga *Turbinaria conoides* [54], and *Pleosporaceae*, a large family within the order Pleosporales that includes numerous marine representatives and recently described strains from the seagrass *Posidonia oceanica* and the jellyfish *Pelagia noctiluca* [55]. One ASV was classified to genus level, *Nigrograna* (also *Pleosporales*), known for broad ecological roles as saprobes, endophytes and pathogens across terrestrial and aquatic environments [56], and one to species level, *Parathyridariella dematiacea*, originally described from the green alga *Flabellia petiolate* [57]. The remaining four *Ascomycota* ASVs were resolved only to phylum level.

Within the *Basidiomycota*, two ASVs were identified as *Pseudotomentella* species, ectomycorrhizal fungi unlikely to associate biologically with seaweeds and detected only at Site A. This suggests terrestrial environmental DNA input, potentially via runoff, consistent with its proximity to human and agricultural activity, further supporting environmental differences between sites. The remaining two *Basidiomycota* ASVs were classified to genus level: *Deconica*, a small genus with no recorded marine isolates [20], and *Vishniacozyma*, a globally distributed, marine-adapted basidiomycetous yeast isolated from seawater, intertidal zones [58] and marine sponges [59].

Only three identified key ASVs were found to significantly differ across algal groups. Despite dominant fungal epiphytic variants across algal samples, differences in community composition are likely determined by subtle differences across multiple fungal taxa [10]. Dominant core operational taxonomic units (OTUs) have been consistently identified across seaweed and seagrass microbiomes [10, 18], with variation attributed to numerous unique, low-abundance OTUs [50]. Here, large differences were noted in the dominant ASVs, even among samples from the same algal species, suggesting the potential influence of stochastic processes, such as random colonization or ecological drift, on fungal community assembly. It is also possible that some of these unassigned dominant strains belong to similar taxonomic groups. Clarifying fungal community patterns between native and invasive macroalgal hosts will require larger sample sizes, inclusion of all unique, low-abundance ASVs, and phylogenetic investigation.

Fungal communities associated with macroalgae are also subject to temporal variability. Within four months, fungal relative abundance was shown to alter from < 1% to circa 30% across samples from five predominant macroalgal species to the Mediterranean coast [60]. As the present study did not assess temporal variation, the findings represent only a snapshot and should not be extrapolated. Furthermore, microbial composition can vary across algal tissue types. Perennial structures such as holdfasts and stipes can host distinct communities compared to ephemeral fronds [10, 19], conceivably because of their distinct chemical ecologies [17]. Thus, results here may reflect holdfast-associated communities only rather than the whole-organism mycobiome.

Limitations related to technical bias are widely acknowledged in genomic studies, and include each step of the sequencing process (DNA extraction methods, primer choice, sequencing platform, and protocols), as well as the bias found in bioinformatics and data analysis. Primer choice, particularly within the ITS region, can introduce taxonomic bias and represents an inherent limitation when a single marker is employed, as in this study. The ITS1 region has been reported to preferentially amplify *Ascomycota*, whereas ITS2 favour *Basidiomycota* [61], potentially skewing apparent community composition. Such primer-associated biases limit accurate assessment of fungal diversity, and the use of alternative or multi-locus primer sets may reduce unassignable sequences and enhance detection of fungal taxa [62]. The widespread presence of unidentified fungal sequences across various marine substrates and environments, however, underscores the significant amount of work still needed to sequence and classify marine fungal species [23]. This is key to the understanding of the function and role of marine fungi to its host and wider ecosystem, and to allow for inter-research comparison.

Exploration of the fungal microbiome of *Sargassum* spp. in their native as well as invasive environment, in conjunction with those of other algal species in close proximity, will provide more insight into the adaptability of the holobiont of invasive and native macroalgae to changing biotic and abiotic factors. This may serve to provide vital insight that contributes to the future management of coastal macroalgal ecosystems, particularly in a changing marine environment.

## Conclusion

Fungal diversity appears to be consistent in the marine environment across small geographic scales. Community composition is probably driven by abiotic factors, but the presence of established, invasive *Sargassum muticum* could act to influence the microbiome of native macroalgal species in its vicinity. Differences in fungal community are likely to be determined by subtle changes across multiple taxa, or the presence of unique, low abundance ASVs.

The macroalgal mycobiome is a complex, dynamic and synergistic relationship between a host’s autogenic secondary metabolites, surface epibionts, and its environment. Continued exploration of the role of macroalgal microbiomes, and their flexibility on exposure to biotic and abiotic factors, is required to understand the magnitude of the impact of this holobiont on invasive success, and subsequently, local and widescale biodiversity.

## Supplementary Information

Below is the link to the electronic supplementary material.

Supplementary Material 1

Supplementary Material 2

## Data Availability

The datasets generated and/or analysed during the current study are provided in the Supplementary Information. Raw sequencing reads and metadata have been deposited in the NCBI Sequence Read Archive (SRA) under the accession number PRJNA1258083.
