# Supplementary material for: Patterns in Marine Fungal Diversity and Community Structure on Native Versus Invasive Macroalgae at a Local Geographic Scale
Source: Microb Ecol. 2026 Apr 23;89(1):122. doi: 10.1007/s00248-026-02781-8 (PMC13243329; doi:10.1007/s00248-026-02781-8)
Supplement: Supplementary file 2 — Supplementary Material 2 [file 248_2026_2781_MOESM2_ESM.docx]

SUPPLEMENTARY INFORMATION

Title**: Patterns in Marine Fungal Diversity and Community Structure on Native versus Invasive Macroalgae at a Local Geographic Scale**

Journal: **Microbial Ecology**

**Elizabeth A.M. Owen**

Marine Centre Wales, School of Ocean Sciences, Bangor University, Menai Bridge, Isle of Anglesey, LL59 5AB, UK.

**Robert I. Griffiths**

Environment Centre Wales, School of Environmental and Natural Sciences, Bangor University, Bangor, LL57 2UW, UK

**Peter N. Golyshin**

Centre for Environmental Biotechnology, School of Environmental and Natural Sciences, Bangor University, Bangor, LL57 2UW, UK

**Tatyana N. Chernikova**

Centre for Environmental Biotechnology, School of Environmental and Natural Sciences, Bangor University, Bangor, LL57 2UW, UK

**Martyn Kurr**

Marine Centre Wales, School of Ocean Sciences, Bangor University, Menai Bridge, Isle of Anglesey, LL59 5AB, UK.

Corresponding author: m.kurr@bangor.ac.uk

Figures (Figure S1)

**Figure S1**

**Fig. S1**  Rarefaction curves showing observed ASV richness as a function of sequencing depth across samples. Samples are coded by host and site: FSA, Fucus serratus (site A); FSB, Fucus serratus (site B); SDA, sediment (site A); SDB, sediment (site B); SMA, Sargassum muticum (site A); SW, seawater. All samples were included in the analysis.

Tables (Table S1- Table S3)

**Table S1** ASV abundance table including taxonomic assignments and read counts for all samples.

**Table S2a** Statistical analysis of mean proportional abundance of individual ASVs between algal groups.

| ASV | Statistical Test | n | Degrees of Freedom | Chi^2^ | F value | p value |
| --- | --- | --- | --- | --- | --- | --- |
| ASV1 | Anova | 26 | 2, 23 |  | 3.390 | 0.05. |
| ASV2 | Anova | 26 | 2, 23 |  | 1.484 | 0.248 |
| ASV3 | Anova | 26 | 2, 23 |  | 3.143 | 0.062 |
| ASV4 | Anova | 26 | 2, 23 |  | 0.464 | 0.634 |
| ASV5 | Anova | 26 | 2, 23 |  | 1.259 | 0.303 |
| ASV7 | Anova | 26 | 2, 23 |  | 0.538 | 0.591 |
| ASV8 | Anova | 26 | 2, 23 |  | 1.296 | 0.293 |
| ASV9 | Anova | 26 | 2, 23 |  | 0.164 | 0.85 |
| ASV10 | Kruskal-Wallis | 26 | 2 | 10.139 |  | 0.006* |
| ASV11 | Kruskal-Wallis | 26 | 2 | 7.304 |  | 0.026* |
| ASV15 | Anova | 26 | 2, 23 |  | 2.084 | 0.147 |
| ASV17 | Kruskal-Wallis | 26 | 2 | 2.250 |  | 0.325 |
| ASV32 | Anova | 26 | 2, 23 |  | 0.744 | 0.486 |
| ASV149 | Anova | 26 | 2, 23 |  | 0.94 | 0.405 |
| ASV150 | Anova | 26 | 2, 23 |  | 0.94 | 0.405 |

**Table S2b** Post hoc tests on individual ASVs showing significance in mean proportional abundance between groups (*Fucus serratus* at site A, *Sargassum muticum* at Site A and *F. serratus* at site B).

| ASV | Post hoc test | Between groups | p value |
| --- | --- | --- | --- |
| ASV1 | Tukey | F. serratus A – F. serratus B  S. muticum A – F. serratus A  S. muticum A – F. serratus B | 0.045*  0.706  0.199 |
| ASV10 | Dunn | F. serratus A – F. serratus B  S. muticum A – F. serratus B | 0.008*  0.008* |
| ASV11 | Dunn | F. serratus A – F. serratus B  S. muticum A – F. serratus B | 0.027*  0.027* |

**Table S3** Analysis performed on diversity indices data taken from studies examining marine fungal diversity to assess for statistical difference in diversity across sites [33, 34].

| Studies of data extraction | Location | Site size | Diversity Index | Data Range | Mean | Statistical test and results. |
| --- | --- | --- | --- | --- | --- | --- |
| This study  Walker and Robincheau (2021)  Nakbi et al. (2023) | North Wales  Gulf of Mexico, U.S.  Monastir Bay, Tunisia | <800m  2,703km  38km | Shannon – Weiner | 0.6 – 2.6  1.5 – 3.0  0.2 – 0.84 | 1.923  2.486  0.569 | ANOVA (df=2, p=0.97) |
| This study  Nakbi et al. (2023) | North Wales  Monastir Bay, Tunisia | <800m  38km | Simpson | 0.43 – 0.92  0.44 - 0.86 | 0.769  0.737 | t-test (t=0.5(6.2), p=0.634) |
